# In vitro modelling of local gene therapy with IL-15/IL-15Rα and a PD-L1 antagonist in melanoma reveals an interplay between NK cells and CD4^+^ T cells

**DOI:** 10.1038/s41598-023-45948-w

**Published:** 2023-11-03

**Authors:** Robin Maximilian Awad, Yannick De Vlaeminck, Fien Meeus, Thomas Ertveldt, Katty Zeven, Hannelore Ceuppens, Cleo Goyvaerts, Magali Verdonck, Gustavo Salguero, Geert Raes, Nick Devoogdt, Karine Breckpot

**Affiliations:** 1 Translational Oncology Research Center (TORC), Laboratory for Molecular and Cellular Therapy (LMCT), Department of Biomedical Sciences (BMWE), Vrije Universiteit Brussel, Laarbeeklaan 103/E, 1090 Brussels, Belgium; 2 In Vivo Cellular and Molecular Imaging Laboratory, Department of Medical Imaging, Vrije Universiteit Brussel, 1090 Brussels, Belgium; 3 Advanced Therapies Unit, Instituto Distrital de Ciencia Biotecnología e Innovación en Salud-IDCBIS, 111611 Bogotá, Colombia; 4 Laboratory of Cellular and Molecular Immunology, Department of Bioengineering Sciences, Vrije Universiteit Brussel, 1050 Brussels, Belgium; 5 Laboratory of Myeloid Cell Immunology, VIB Center for Inflammation Research, 1050 Brussels, Belgium; 6 Laboratory of Dendritic Cell Biology and Cancer Immunotherapy, VIB Center for Inflammation Research, 1050 Brussels, Belgium

**Keywords:** Innate immune cells, Lymphocytes, Tumour immunology, Cancer, Immunology, Molecular biology, Oncology

## Abstract

Blockade of the immune checkpoint axis consisting of programmed death-1 (PD-1) and its ligand PD-L1 alleviates the functional inhibition of tumor-infiltrating lymphoid cells yet weakly induces their expansion. Exogenous cytokines could further expand lymphoid cells and thus synergize with αPD-L1 therapy. However, systemic delivery of most cytokines causes severe toxicity due to unspecific expansion of immune cells in the periphery. Here, we modelled local delivery of cytokines and αPD-L1 therapeutics to immune cell-containing in vitro melanoma tumors. Three-dimensional tumor models consisting of 624-MEL cells were co-cultured with human peripheral blood lymphoid cells (PBLs) in presence of the cytokines IL-2, IL-7, IL-15, IL-21 and IFN-γ. To model local gene therapy, melanoma tumors were modified with lentiviral vectors encoding IL-15 fused to IL-15Rα (IL-15/IL-15Rα) and K2-Fc, a fusion of a human PD-L1 specific single domain antibody to immunoglobulin (Ig)G1 Fc. To evaluate the interplay between PBL fractions, NK cells, CD4^+^ T cells or CD8^+^ T cells were depleted. Tumor cell killing was followed up using real time imaging and immune cell expansion and activation was evaluated with flow cytometry. Among the tested cytokines, IL-15 was the most potent cytokine in stimulating tumor cell killing and expanding both natural killer (NK) cells and CD8^+^ T cells. Gene-based delivery of IL-15/IL-15Rα to tumor cells, shows expansion of NK cells, activation of NK cells, CD4^+^ and CD8^+^ T cells, and killing of tumor spheroids. Both NK cells and CD8^+^ T cells are necessary for tumor cell killing and CD4^+^ T-cell activation was reduced without NK cells. Co-delivery of K2-Fc improved tumor cell killing coinciding with increased activation of NK cells, which was independent of bystander T cells. CD4^+^ or CD8^+^ T cells were not affected by the co-delivery of K2-Fc even though NK-cell activation impacted CD4^+^ T-cell activation. This study demonstrates that gene-based delivery of IL-15/IL-15Rα to tumor cells effectively mediates anti-tumor activity and sensitizes the tumor microenvironment for therapy with αPD-L1 therapeutics mainly by impacting NK cells. These findings warrant further investigation of gene-based IL-15 and K2-Fc delivery in vivo.

## Introduction

Tumor-infiltrating lymphoid cells with cytolytic capacity such as natural killer (NK) cells and CD8^+^ T cells are key in controlling tumor growth. Therefore, these immune effector cells have gained a lot of attention in cancer immunotherapy^1,2^. In solid tumors, NK cells and CD8^+^ T cells often display reduced cytotoxicity due to a state of exhaustion. This dysfunctional status can be remedied by blocking signaling via inhibitory receptors such as programmed death-1 (PD-1, CD279)^3^.

Immunotherapy with monoclonal antibodies targeting PD-1 and its ligand PD-L1 (B7-H1, CD274) transformed the immune oncology landscape and became a mainstay therapy option for many malignancies^4^. Disrupting the interaction between PD-1 and PD-L1 recovers the cytolytic function of immune cells such as T cells and NK cells. However, despite encouraging results in subsets of patients, the majority of patients does not respond to the therapy or only remains temporarily progression-free after initiating immune checkpoint therapy^5^. It is contended that the inability to expand cytolytic tumor-infiltrating lymphoid cells is a major contributing factor to therapy failure or tumor relapse^6^. Therefore, additional approaches to expand cytolytic immune cells and as such reshape the tumor microenvironment (TME) are needed to improve the outcome of immune checkpoint therapy. In this regard, cytokine therapy has been studied^7^.

Various cytokines have the potential to enhance anti-tumor immune responses by expanding NK cells and T cells^8^. Common γ-chain (γc) cytokines such as interleukin (IL)-2, IL-7, IL-15 and IL-21 and other cytokines such as interferon (IFN)-γ stimulate the expansion and survival of cytolytic lymphoid cells yet can have a varying impact on their functionality^9^. Therefore, studies into exogenous cytokines and how these expand lymphoid cells and thus synergize with αPD-L1 therapy are being performed. Most attention has been given to IL-15^10^, a pleiotropic cytokine produced by myeloid and stromal cells^11,12^, that expands and reinvigorates NK cells and CD8^+^ T cells^10^. Due to these properties, IL-15 therapy became attractive in immune oncology and is currently under clinical investigation. As systemic delivery of most cytokines causes severe toxicity due to unspecific expansion of immune cells in the periphery, strategies to locally deliver cytokines are under investigation^13^. Targeting IL-15 to tumors has been achieved using antibodies to tumor-associated antigens^14–17^. However, the full potential might not be achieved as on-target yet off-tumor effects may occur due to the high affinity of IL-15 to its receptor and as tumor cells might internalize those fusion proteins and limit their targeting to T cells.

Intratumoral immunotherapy is receiving increasing attention^18^, opening avenues for a gene-based approach to cytokine and immune checkpoint therapy. We previously developed K2, a single domain antibody (sdAb) fragment that competes with PD-1 for binding to PD-L1^19,20^. We showed that gene-based expression of K2-Fc, a fusion of K2 to human IgG1 Fc, in melanoma spheroids efficiently blocked the interaction of PD-1 with PD-L1 and that especially Fc-mediated effector functions substantially contributed to immune cell activation and tumor cell killing^21^. Building on these data, we explored whether gene-based delivery of IL-15 to tumor cells can evoke anti-tumor immune responses and can potentiate the immune stimulatory effects of K2-Fc.

## Methods

### Reagents

Recombinant IL-2 (Peprotech, London, UK), IL-7 (ImmunoTools, Friesoythe, Germany), IL-13 (ImmunoTools), IL-15 (Peprotech), IL-21 (Peprotech), IFN-γ (Abcam, Cambridge, UK) and CD3/CD28 beads (Miltenyi Biotec, Bergisch Gladbach, Germany) were used for stimulation where indicated. Brilliant Violet (BV) 421 rat anti-human IgG-Fc antibodies (clone M1310G05, BioLegend, USA) were used to stain K2-Fc and R3-Fc on PD-L1^+^ 624-MEL cells in flow cytometry. Expression of IL-15 by green fluorescent protein (GFP)^+^ 624-MEL cells was verified using allophycocyanin (APC)-conjugated mouse anti-human IL-15 antibodies (clone 34559, Thermo, USA). Antibodies used for characterization of immune cells were BV605-conjugated anti-CD3 (clone UCHT1, BioLegend), peridinin-chlorophyll (PERCP)-cyanine (CY) 5.5-conjugated anti-CD4 (clone RPA-T4, BioLegend), BV421-conjugated anti-CD8 (clone HB15e), phycoerythrin (PE)-CY7-conjugated anti-CD56 (clone MEM-188, BioLegend), Alexa Fluor (AF) 700-conjugated anti-CD45 (clone H130, BioLegend), APC-Fire 750-conjugated anti-CD16 (clone 3G8, BioLegend), fluorescein isothiocyanate (FITC)-conjugated anti-PD-1 (clone A17188B, BioLegend), APC-conjugated anti-CD134 (clone ACT35, BioLegend), and PE-conjugated anti-CD137 (clone 4B4-1, BioLegend). Zombie Aqua (BioLegend) was used to discriminate between live and dead cells. Anti-CD4, CD8 or CD19 magnetic beads (Miltenyi) were used for selection and depletion of CD4^+^ and CD8^+^ T cells.

### Cell lines

The HLA-A2 positive cell line 624-MEL was obtained from S.L. Topalian (National Cancer Institute, Baltimore, MD, USA). This cell line and its GFP^+^^21^ or PD-L1^+^^19^ variants, were cultured in Roswell Park Memorial Institute (RPMI) 1640 medium (Sigma-Aldrich) supplemented with 10% FetalClone I serum (FCI, Cytiva, Marlborough, USA), 2 mM L-glutamine, 100U/mL penicillin, 100 μg/mL streptomycin, 1 mM sodium pyruvate, and non-essential amino acids (Sigma-Aldrich). The HEK293T cell line was obtained from the American Type Culture Collection and was cultured in Dulbecco's Modified Eagle Medium (DMEM, Sigma-Aldrich) supplemented with 10% fetal bovine serum (FBS, Harlan) and 100 U/mL penicillin, 100 µg/mL streptomycin and 2 mM L-glutamine.

### Peripheral blood lymphocytes

Peripheral blood lymphocytes (PBLs) from HLA-A2 positive donors were collected as previously described^21^. Briefly, leukapheresis (Spectra Optia®, TerumoBCT) was performed on consenting healthy donors (Ethical license B.U.N. 143201420996) in accordance with the relevant guidelines and regulations. The leukapheresis product was processed via an elutriation procedure to collect monocytes (Elutra®, TerumoBCT). The remaining PBLs were counted and cryopreserved in 5% DMSO-containing cryopreservation medium (CryoStor® CS5, Biolife Solutions) till further use.

To study the contribution of NK cells to the observed effects, PBLs were depleted from CD19^+^ cells using anti-CD19 magnetic beads to enhance purity of NK, CD4^+^ and CD8^+^ T cells within the PBLs. Subsequently, CD4^+^ and CD8^+^ T cells were depleted with respectively anti-CD4 and anti-CD8 magnetic beads. To study the contribution of T cells to the observed effects, PBLs were selected for CD4^+^ and CD8^+^ T cells using anti-CD4 and anti-CD8 magnetic beads. Depletions and selections were performed according to the manufacturer’s instructions.

### Cloning of lentiviral transfer plasmids

The pHR’ lentiviral transfer plasmid was used as a template to produce a lentiviral transfer plasmid that encodes IL-15/IL-15Rα. The construct contains a murine Igκ-signal peptide, the IL-15 sequence (NCBI Reference Sequence: NP_000576.1) and was connected via a P2A self-cleaving site to a Igκ-signal peptide followed by the IL-15Rα sequence (NCBI Reference Sequence: NP_002180.1) and a hemagglutinin (HA)-tag.

The gene fragments were designed in silico using SnapGene (Dotmatics, USA) and subsequently codon optimized using the IDT codon optimization tool. We added 3’ and 5’ sequences that were homologous to the pHR’ plasmid insertion site and ordered the synthetic gene fragments (gBlocks, Integrated DNA Technologies, USA). The transfer plasmid was opened with restriction enzymes EcoRI and BamHI (Thermo Fisher Scientific, USA) and the gBlocks were ligated using the NEBuilder HiFi DNA Assembly mix (New England Biolabs, USA). Competent bacteria (New England Biolabs) were transformed, expanded and plasmid DNA was isolated using the GeneJET Plasmid Maxiprep Kit (Thermo Fisher Scientific). Quantities of plasmid DNA were evaluated using a spectrophotometer.

The lentiviral transfer plasmids encoding K2-Fc or R3-Fc^21^, an antibody-variant of the nanobody that binds the idiotype of 5T2 myeloma cells (R3B23)^22^, were previously described.

### Lentiviral vector production

Lentiviral vectors were produced as described earlier^23^. Briefly, 293T cells were transfected with the envelope plasmid pMD.G, the packaging plasmid pCMVΔR8.9 and the transfer plasmid pHR’ encoding the genes of interest with polyethyleneimine. Lentiviral supernatants were harvested at 48 and 72 h.

### Transduction of tumor cells with lentiviral vectors

624-MEL cells that express GFP were seeded in a six-well tissue culture plate (Thermo Fisher Scientific) at 10^5^ cells per well in 2 mL culture medium. Twenty-four hours later, supernatants were removed from the adherent cells and replaced by a transduction cocktail containing lentiviral vectors that were pre-incubated for 15 min with 1 µg/mL protamine sulfate. The transduction cocktail was removed 3 days later when cells were transferred to a larger recipient for cell expansion.

### Enzyme-linked immunosorbent assay

Supernatants of GFP^+^ 624-MEL cells that were lentivirally transduced to express IL-15/IL-15Rα were subjected to the human IL-15 enzyme-linked immunosorbent assay (ELISA) Kit (ab218266) (Abcam). The ELISA was performed according to the manufacturer’s recommendations.

### Spheroid tumor cell killing assay

624-MEL cells that express GFP and that were lentivirally transduced to express the gene of interest, were seeded at 10^4^ cells in 100 µL culture medium in a 96-well ultra-low attachment plate (Greiner Bio-One, Frickenhausen, Germany). After 72 h, 3 × 10^5^ PBLs in 100 µL culture medium were added. In case non-modified 624-MEL-GFP cells were used, recombinant proteins were added at the following concentration: IL-2: 100 ng/mL, IL-7: 100 ng/mL, IL-13: 20 ng/mL, IL-15: 10 ng/mL, IL-21: 100 ng/mL and IFN-γ: 200 ng/mL. As a positive control, beads coated with anti-CD3 and anti-CD28 antibodies (Miltenyi) were added according to the manufacturer’s instructions to the PBLs to activate T cells. The plate was transferred to the IncuCyte ZOOM live imaging system (EssenBio) to image the green fluorescent area, a measure of the tumor cell number, in real-time. A picture at a 4-times magnification was taken every two hours for five days or longer. Images were processed and analyzed using the IncuCyte Zoom software (Essen Bioscience, USA). The area under the curve (AUC) of the green fluorescent confluence was calculated using GraphPad Prism (Dotmatics) and used to calculate the specific cell killing with the following formula: 1 - AUC/AUC_untreated_ × 100. In parallel, cultures were set up to analyze immune cell activation at the time that the tumor size was visibly reduced or followed up daily for kinetic analysis.

### Immune cell subsets expansion assay

624-MEL-GFP cells, lentivirally transduced to express the gene of interest, were seeded at 5 × 10^4^ cells in 100 µL culture medium in a 96-well ultra-low adherence plate (Greiner Bio-One). After 72 h, 3 × 10^5^ PBLs in 100 µL culture medium were added. Immune cells were collected after 5, 7 and 10 days post co-culture and analyzed with flow cytometry.

### Flow cytometry

Flow cytometry was performed to study the expression of IL-15, K2-Fc or R3-Fc after lentiviral transduction of 624-MEL-GFP cells as well as to study the immune cell composition and activation of PBLs after co-culture with tumor cells. Cell staining procedures were performed as described previously^24^. Cells were acquired on the LSR Fortessa (BD Biosciences, Franklin Lakes, NJ, USA), and cell analysis was performed using the FlowJo software (BD Biosciences).

### Statistical analysis

Statistical analyses were performed either by t-test, matched one-way ANOVA with Geisser-Greenhouse correction and mixed effects model with multiple comparisons or by matched two-way ANOVA with Tukey’s multiple comparisons test. The analyses were performed using GraphPad Prism v.9.3.1. Statistical significance was indicated with * for *p* < 0.05; ** for *p* < 0.01, *** for *p* < 0.001, **** for *p* < 0.001 and ns for non-significant.

### Ethics approval and consent to participate

Ethics approval for experiments with consenting healthy donors was obtained from the Medical ethics commission of UZ Brussel and the VUB (ethical license B.U.N. 143201420996). All methods were performed in accordance with the relevant guidelines and regulations. Informed consent was obtained from all subjects and/or their legal guardian(s).

## Result

### IL-15 stimulates tumor cell killing and modulates immune cell composition and activation

**Figure 1:**
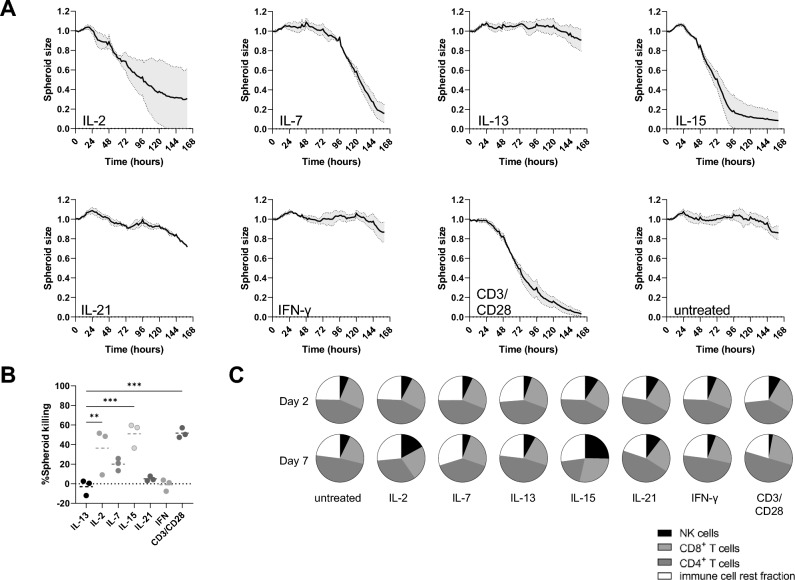
IL -15 stimulates tumor cell killing and modulates the immune cell composition. (**A**) Relative spheroid cell killing as measured as reduction in GFP^+^ area over time using the IncuCyte Zoom live cell imaging system for spheroids generated with 624-MEL-GFP cells and treated with the indicated cytokines as mean ± SEM (n = 3). (**B**) Percentage of spheroid killing for the different conditions. Each dot represents the result of an independent experiment, while the dotted line shows the mean of these experiments (n = 3). (**C**) Distribution of cell subsets within PBLs, defined as CD3^+^ CD4^+^ and CD3^+^ CD8^+^ T cells, CD3^-^ CD56^+^ NK cells and cells that are not identified by CD3 or CD56 (rest fraction) for the indicated conditions, 2 and 7 days after the start of the co-culture (n = 3). The number of asterisks in the figure indicates the statistical significance as follows: **p < 0.01, ***p < 0.001.

We used a 3D cell killing assay to study the ability of recombinant cytokines to enhance immune-mediated destruction of melanoma cells. We studied the γc cytokines IL-2, IL-7, IL-15 and IL-21 or IFN-γ, as these cytokines have been shown to stimulate anti-tumor immunity^9^. We added these cytokines to co-cultures containing 624-MEL-GFP tumor spheroids and PBLs. We used CD3/CD28 T-cell activating beads and IL-13 as respectively positive and negative controls. We observed that addition of T-cell activating beads, IL-2, IL-7 and IL-15 but not IL-13, IL-21 or IFN-γ decreased the spheroid size, measured as a decrease in green fluorescent area, with distinct kinetics between the cytokines (Fig. 1A). However, only conditions containing T-cell activating beads, IL-2 or IL-15 showed statistically significant 624-MEL-GFP tumor spheroid killing (Fig. 1B). We collected PBLs from the 3D cell killing assay at 2 and 7 days of culture and studied the cell composition in multicolor flow cytometry (Supplementary Fig. 1A). After 2 days, we did not observe significant differences in the composition of the PBLs co-cultured with tumor cells in the presence of cytokines or T-cell activator beads (Fig. 1C). However, after 7 days we observed an altered cell composition in co-cultures supplemented with IL-2, IL-7 and IL-15. The fraction of NK cells and CD8^+^ T cells was most prominent in the condition supplemented with IL-15, while the fraction of CD4^+^ T cells decreased in these cultures (Fig. 1C). Subsequently, we investigated the expression of PD-1, CD134, CD137 and CD16 on NK cells, CD4^+^ and CD8^+^ T cells, as these markers correlate with early immune cell activation^21,25–27^. IL-15 significantly increased the expression of CD134 and CD137 on NK cells and the expression of PD-1 on T cells (Supplementary Fig. 1B).

### Genetic delivery of IL-15 stimulates immune cell-mediated tumor cell killing and modulates the immune cell composition and activation

**Figure 2:**
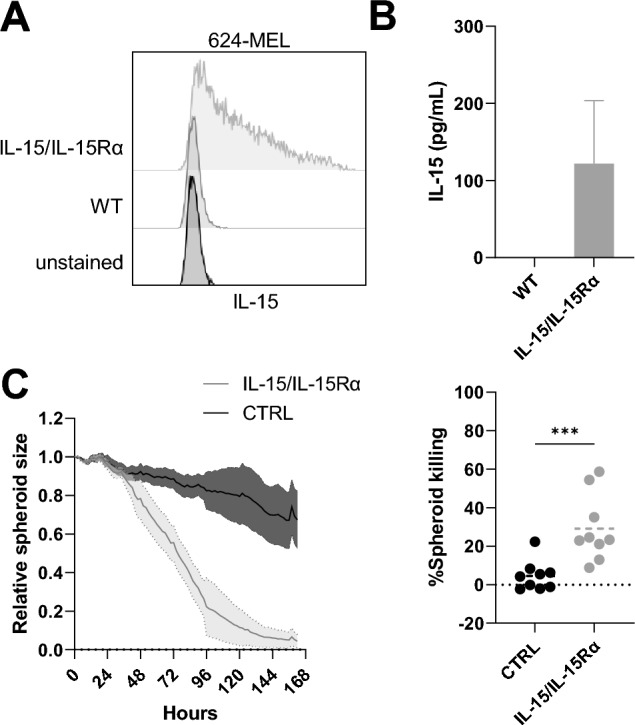
Genetic IL-15-delivery stimulates immune cell-mediated tumor cell killing. (**A**) Flow cytometry histograms showing the production of IL-15 after lentiviral transduction of 624-MEL-GFP cells. (**B**) Concentration of IL-15 in supernatants of 624-MEL-GFP cells that were lentivirally transduced to express IL-15. (**C**) The graph on the left shows relative spheroid cell killing over time as measured using the IncuCyte Zoom live cell imaging system for spheroids generated with 624-MEL-GFP cells that were lentivirally transduced with IL-15 encoding or control vector as mean ± SEM (n = 9). The graph on the right shows percentages of spheroid killing. Each dot represents the result of an independent experiment, while the line shows the mean of these experiments (n = 9). The number of asterisks in the figure indicates the statistical significance as follows: ***p < 0.001.

The genetic code of IL-15 and its co-receptor IL-15Rα were cloned into a lentiviral transfer plasmid for the production of lentiviral vectors. It was shown earlier that association of IL-15 with IL-15Rα results in the formation of a complex with improved biologic activity^28^. Lentiviral vectors were used to transduce 624-MEL-GFP cells. We confirmed the expression of IL-15/IL-15Rα by these cells using flow cytometry (Fig. 2A). To measure the quantity of secreted IL-15 we harvested supernatants from these cells and performed ELISA. IL-15 was detected in the supernatants of transduced 624-MEL-GFP cells (Fig. 2B). Next we evaluated whether the transgenic IL-15/IL-15Rα expression was sufficient to induce tumor cell killing. To this end, tumor spheroids were made from lentivirally modified 624-MEL-GFP cells and co-cultured with PBLs. Tumor cell killing was monitored microscopically in real-time. We observed that immune cell-mediated tumor cell killing was facilitated when IL-15/IL-15Rα was produced by 624-MEL-GFP cells (Fig. 2C).

**Figure 3:**
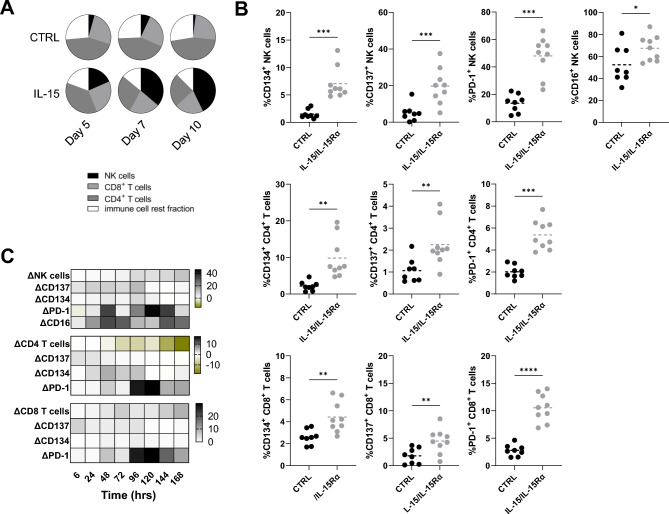
Genetic IL-15-delivery modulates the immune cell composition and activation. (**A**) Distribution of cell subsets within PBLs, defined as CD3^+^ CD4^+^ and CD3^+^ CD8^+^ T cells, CD3^−^ CD56^+^ NK cells and cells that are not identified by CD3 or CD56 (rest fraction) for the indicated conditions, 5, 7 and 10 days after the start of the co-culture (n = 3). (**B**) Expression of activation markers CD134, CD137, PD-1 and CD16 on CD3^-^ CD56^+^ NK cells (upper panel), CD3^+^ CD4^+^ T cells (middle panel) and CD3^+^ CD8^+^ T cells (lower panel) measured in flow cytometry at 48 h in co-culture (n = 9). (**C**) Heatmap showing the differential modulation of immune cell composition and activation marker compared to the expression detected in the control condition during a week in co-culture (n = 3). The number of asterisks in the figure indicates the statistical significance as follows: *p < 0.05, **p < 0.01, ***p < 0.001, ****p < 0.0001.

Like recombinant IL-15, transgenic IL-15/IL-15Rα produced by 624-MEL-GFP cells expanded the NK cell compartment dramatically (Fig. 3A). However, little CD8^+^ T-cell expansion was observed (Fig. 3A). Nevertheless, NK cells, CD4^+^ T cells and CD8^+^ T cells were activated at early time points (48 h) in the presence of IL-15/IL-15Rα, evidenced by expression of CD134, CD137, PD-1 and in the case of NK cells also CD16 (Fig. 3B). Therefore, we studied how IL-15/IL-15Rα influences the kinetics of immune cell composition and activation marker expression during one week upon the start of co-culture. After 72 h NK and CD8^+^ T cells gradually expanded, coinciding with a decrease of CD4^+^ T cells. The expression of CD134 and CD137 was upregulated on CD4^+^ or CD8^+^ T cells and NK cells within the first 96 h after which the expression was no longer detected. In contrast, PD-1 expression was highly upregulated in all immune cell subsets from 96 h onwards. On NK cells, the expression of CD16 was upregulated as early as 24 h after culture, remaining high throughout the entire period of analysis (168 h) (Fig. 3C).

### Cytolytic immune cells play a major role in IL-15/IL-15Rα mediated tumor cell killing, though immune cell profiling reveals an interplay between NK cells and CD4^+^ T cells

**Figure 4:**
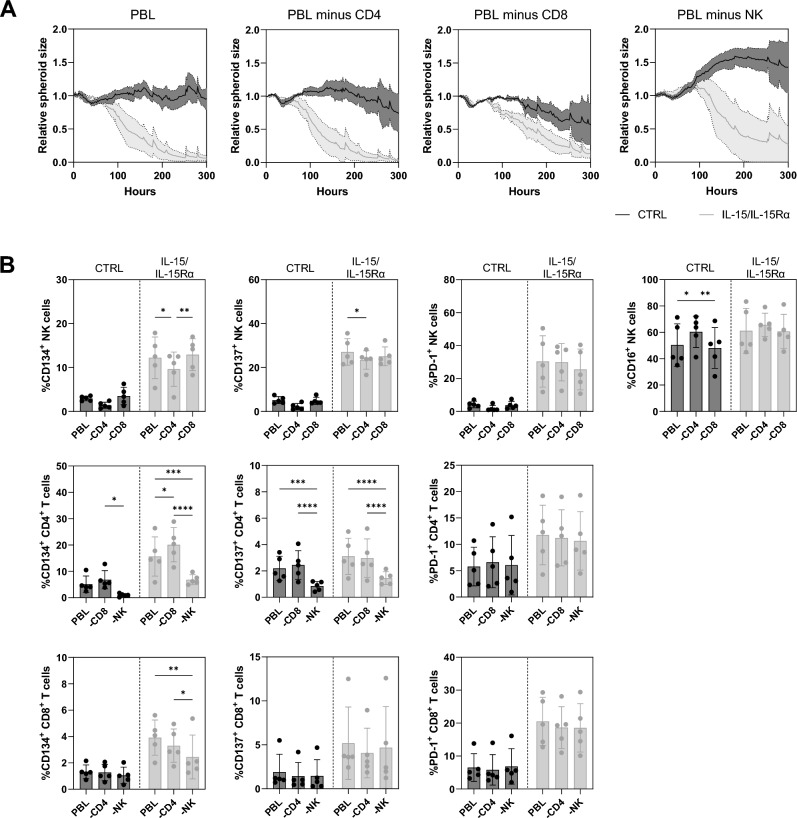
NK cells and CD8^+^ T cells are responsible for IL-15 mediated tumor control and tumor cell killing. (**A**) Reduction in GFP^+^ area over time for three independent experiments, with 624-MEL tumor cells that produce IL-15 (gray lines) and 624-MEL cells that do not produce IL-15 (black lines) and that were co-cultured with PBLs that were respectively not depleted, CD4 depleted, CD8 depleted or innate immune cell depleted. The GFP area was measured using the IncuCyte Zoom live cell imaging system (n = 3). (**B**) Expression of activation markers CD134, CD137, PD-1 and CD16 on CD3^-^ CD56^+^ NK cells (upper row), CD3^+^ CD4^+^ T cells (middle row) and CD3^+^ CD8^+^ T cells (lower row) measured in flow cytometry at 72 h in co-culture (n = 5). The number of asterisks in the figure indicates the statistical significance as follows: *p < 0.05, **p < 0.01, ***p < 0.001, ****p < 0.0001.

We showed that genetically delivered IL-15/IL-15Rα acts on NK cells, CD4^+^ and CD8^+^ T cells during PBL-mediated tumor cell killing. To study whether these effects result from an interplay between the different cell subsets, we performed the 3D killing assay upon depletion of CD4^+^ T cells, CD8^+^ T cells or NK cells. After depletion of CD4^+^ T cells there are no significant differences in tumor cell killing compared to when using nondepleted PBLs, suggesting that CD4^+^ T cells are dispensable for IL-15 mediated tumor cell killing (Fig. 4A). CD8^+^ T cell depletion changed the slope in the IL-15/IL-15Rα condition, resulting in reduced tumor cell killing. Interestingly, the tumor spheroid killing in the control condition was even stronger in absence of CD8^+^ T cells. When NK cells were depleted, the tumor control was impaired resulting in tumor spheroid growth unless IL-15/IL-15Rα was present in the culture. IL-15/IL-15Rα reverted the tumor growth effect and mediated T-cell driven tumor spheroid killing (Fig. 4A).

We collected the immune cells from the co-cultures during tumor cell killing and evaluated their activation marker expression (Fig. 4B). Immune activation was confirmed in conditions containing IL-15/IL-15Rα. NK cells upregulated CD134, CD137, PD-1 and CD16 in the presence of IL-15/IL-15Rα and depletion of CD4^+^ T cells yet not CD8^+^ T cells impacted on the expression of CD134, CD137 and CD16, while PD-1 expression was unaffected. Expression of the activation markers CD134 and CD137 on CD4^+^ T cells was significantly reduced in cultures lacking NK cells, while the expression of PD-1 was again unaffected. These results suggest an interplay between NK cells and CD4^+^ T cells. Depletion of CD8^+^ T cells significantly increased the expression of CD134 on CD4^+^ T cells, which was unexpected. Similarly unexpected was the little impact of depleting CD4^+^ T cells or NK cells on the activation of CD8^+^ T cells with only a significant decrease in expression of CD134 in cultures containing IL-15/IL-15Rα (Fig. 4B).

### Gene-based co-delivery of IL-15/IL-15Rα and K2-Fc improves tumor cell killing and immune cell activation

**Figure 5:**
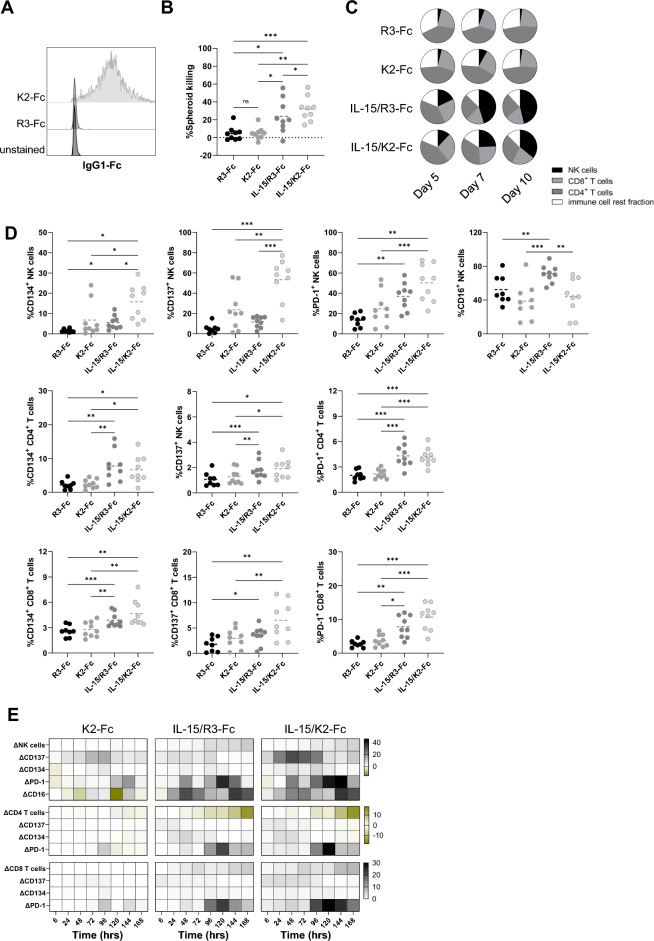
Gene-based co-delivery of IL-15 and K2-Fc improves tumor cell killing. (**A**) The histogram shows 624-MEL PD-L1^+^ cells that were incubated with supernatants derived from 624-MEL-GFP cells that were transduced with lentiviral vectors encoding R3-Fc or K2-Fc. (**B**) Percentage of spheroid killing for the indicated conditions. Each dot represents the result of an independent experiment, while the dotted line shows the mean of these experiments (n = 9). (**C**) Distribution of cell subsets within PBLs, defined as CD3^+^ CD4^+^ and CD3^+^ CD8^+^ T cells, CD3^-^ CD56^+^ NK cells and cells that are not identified by CD3 or CD56 (rest fraction) for the indicated conditions, 5, 7 and 10 days after the start of the co-culture (n = 3). (**D**) Expression of activation markers CD134, CD137, PD-1 and CD16 on CD3^-^ CD56^+^ NK cells (upper row), CD3^+^ CD4^+^ T cells (middle row) and CD3^+^ CD8^+^ T cells (lower row) measured in flow cytometry at 48 h (n = 9). (**E**) Heatmap showing the differential modulation of immune cell composition and activation marker compared to expression in the control condition in function of the time (n = 3). The number of asterisks in the figure indicates the statistical significance as follows: *p < 0.05, **p < 0.01, ***p < 0.001, ****p < 0.0001.

Since IL-15/IL-15Rα produced by tumor cells upregulated PD-1 and CD16 on NK cells and PD-1 on T cells (Figs. 3B,C and 4B) and since NK cells and CD8^+^ T cells were identified as drivers for immune-mediated tumor cell killing (Fig. 4A), we evaluated whether enhancing the activity of these cells by avoiding PD-1 receptor signaling and stimulating CD16 could facilitate tumor cell killing. We generated lentiviral vectors encoding K2-Fc or R3-Fc (control) and used them to transduce 624-MEL-GFP cells. To verify K2-Fc and R3-Fc expression, we subjected the cells’ supernatants to binding studies on PD-L1 transduced 624-MEL cells. We showed that K2-Fc was found in the supernatant by demonstrating binding on PD-L1^+^ 624-MEL cells (Fig. 5A). Next, we mixed 624-MEL-GFP cells that were modified to express K2-Fc or R3-Fc at a 1:1 ratio with 624-MEL-GFP cells or 624-MEL-GFP cells that expressed IL-15/IL-15Rα. The resulting tumor spheroids were co-cultured with PBLs, showing tumor cell killing when IL-15/IL-15Rα was produced by the tumor cells. The level of tumor cell killing was significantly increased in the presence of K2-Fc (Fig. 5B). Analyses of the immune cell composition in these spheroids confirmed that IL-15/IL-15Rα was able to increase the fraction of NK cells. However, when K2-Fc was simultaneously produced, this increase lagged behind and was slightly diminished (Fig. 5C).

Analysis of the activation state of PBLs during tumor cell killing was evaluated. Figure 5D shows that NK cells display increased expression of CD134, CD137 and PD-1 at 48 h as a result of IL-15/IL-15Rα or K2-Fc alone, which was stimulated further when IL-15/IL-15Rα and K2-Fc were combined. Though not significant, the presence of K2-Fc reduced the detection of CD16 on NK cells. On CD4^+^ and CD8^+^ T cells, IL-15/IL-15Rα but not K2-Fc upregulated CD134, CD137 and PD-1 at 48 h of co-culture. Simultaneous production of K2-Fc and IL-15/IL-15Rα did not increase the expression of these activation markers (Fig. 5D). Next we evaluated whether dual expression of IL-15 and K2-Fc impacted the kinetics of immune cell expansion and CD134, CD137, PD-1 and CD16 expression during tumor cell killing (Fig. 5E). When compared to R3-Fc, K2-Fc had little impact on the immune cell composition though showing upregulation of CD137 between 72 and 96 h and downregulation of CD16 at various time points on NK cells. K2-Fc also stimulated the expression of PD-1, mostly on NK cells and CD8^+^ T cells. Conditions containing IL-15/IL-15Rα and R3-Fc mirrored the results obtained with IL-15/IL-15Rα alone (Figs. 3C, 5E). Likewise, conditions containing IL-15/IL-15Rα and K2-Fc were largely similar to those containing IL-15/IL-15Rα alone with the exception of markedly increased expression of CD137 on NK cells and PD-1 on CD8^+^ T cells from 96 h onwards (Figs. 3C, 5E).

### NK-cell activation in response to IL-15/IL-15Rα and K2-Fc does not depend on CD4^+^ or CD8^+^ T cells, while both T-cell subsets require NK cells for full activation

**Figure 6:**
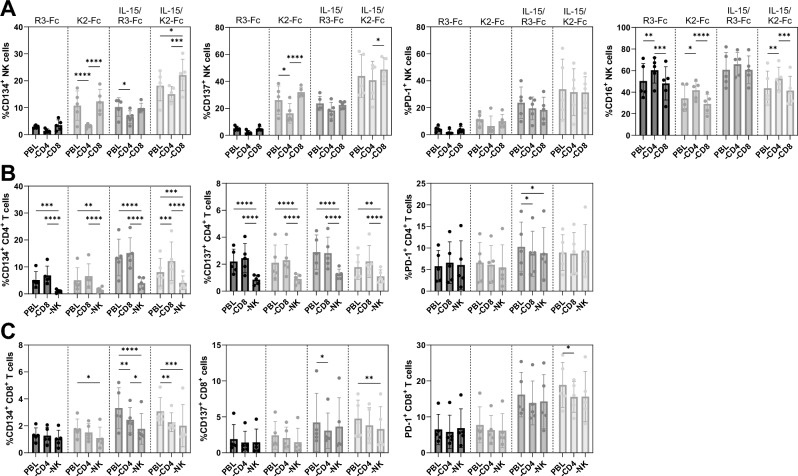
NK cells and CD8^+^ T cells are responsible for IL-15 mediated tumor control and tumor cell killing. Expression of the activation markers CD134, CD137, PD-1 and CD16 on (**A**) CD3^-^ CD56^+^ NK cells after CD4 and CD8 cell depletion (**B**) CD3^+^ CD4^+^ T cells after CD8 and innate immune cell depletion and (**C**) CD3^+^ CD8^+^ T cells after CD4 and innate immune cell depletion measured in flow cytometry at 72 h co-culture (n = 5). The number of asterisks in the figure indicates the statistical significance as follows: *p < 0.05, **p < 0.01, ***p < 0.001, ****p < 0.0001.

Having established that NK cells and CD4^+^ T cells affect each other’s activation status (Fig. 4B), we studied the impact of depleting NK cells, CD4^+^ or CD8^+^ T cells when both IL-15/IL-15Rα and K2-Fc were produced. Figure 6A shows that the expression of CD134 and CD137 on NK cells at 48 h of co-culture was induced by K2-Fc alone and was significantly reduced upon CD4^+^ T-cell depletion. However, when K2-Fc was co-delivered with IL-15/IL-15Rα, CD134 and CD137 expression were upregulated to a similar extent as observed without CD4^+^ T-cell depletion (Fig. 6A). On CD4^+^ T cells, the expression of CD134 and CD137 was downregulated in absence of NK cells and their expression could not be rescued by IL-15/IL-15Rα and/or K2-Fc (Fig. 6B). These data corroborate the interaction between NK cells and CD4^+^ T cells yet highlight that under highly NK-cell stimulatory conditions interactions with CD4^+^ T cells are not required for full NK-cell activation. In contrast, activated NK cells are key to CD4^+^ T-cell activation. The impact of depleting NK cells or CD4^+^ T cells on the activation of CD8^+^ T cells was subtle, as expected based on the results of Fig. 4B, showing mainly downregulation of CD134 when NK cells were depleted in conditions containing K2-Fc and/or IL-15/IL-15Rα. Likewise, depletion of CD4^+^ T cells in conditions containing IL-15/IL-15Rα or both IL-15/IL-15Rα and K2-Fc showed a reduced expression of CD134 on CD8^+^ T cells, though similar effects were not observed for CD137 (Fig. 6C).

## Discussion

In this in vitro study, we demonstrated that genetically delivered IL-15/IL-15Rα modulates the composition and activation of melanoma surrounding immune cells and elicits immune cell-mediated anti-tumor immune responses. Moreover, we demonstrated that gene-based IL-15 delivery could sensitize the tumor microenvironment for immune checkpoint therapy with K2-Fc. The tumor-infiltrating lymphocyte composition and activation are important prognostic factors in melanoma and are suggested to be potential biomarkers for response to immune checkpoint therapy^29,30^. We observed that exogenously and genetically administered IL-15 shape the immune cell compartments differently. While exogenously added IL-15 expanded CD8^+^ T cells and NK cells, in situ produced IL-15 selectively expanded NK cells. It has been shown that naïve CD4^+^ T cells, CD8^+^ T cells and NK cells require different amounts of IL-15 to expand^31^, therefore the picomolar amounts of IL-15 produced by the tumor cells upon lentiviral modification might be insufficient to promote CD8^+^ T cell expansion. Regarding lymphocyte activation, gene-based IL-15 delivery was able to activate lymphocytes as characterized by upregulation of the expression markers CD134, CD137, PD-1 and CD16. IL-15 is known to stimulate the expression of immune checkpoint molecules such as PD-1 and PD-L1^32,33^. We demonstrated that gene-based IL-15 delivery efficiently drives immune cell-mediated melanoma cell killing. Moreover we found that NK cells or CD8^+^ T cells, but not CD4^+^ T cells, were required for tumor cell killing. Similarly, it has been shown that NK cells, CD8^+^ T cells and γδ T cells exhibit cytolytic potential upon IL-15 stimulation in various tumor models^31,34–36^.

Treatment with IL-15 as monotherapy has been shown ineffective due to immune checkpoints and due to the lack of tumor-specific targeting of NK cells^37^. Moreover, it has been shown that IL-15 expanded NK cells can improve the therapeutic efficacy of anti-tumor antibodies through antibody-dependent cellular cytotoxicity (ADCC)^38,39^. Here we found that K2-Fc, an antibody derivative that blocks the PD-1:PD-L1 axis and enables enhanced ADCC, could augment IL-15/IL-15Rα-mediated melanoma cell killing and could synergistically improve CD8^+^ T cell and NK cell activation. These findings are in line with recent studies wherein anti-PD-L1 immunotherapy could be improved through IL-15 treatment in various preclinical tumor models including melanoma^33,40^. Remarkably, without IL-15/IL-15Rα, K2-Fc alone had modest impact on most lymphocyte activation markers, besides CD137 on NK cells, and was not able to drive anti-tumor immune responses profoundly. Therefore, the data presented here suggest that local delivery of IL-15 might convert patients that are irresponsive to anti-PD-L1 therapy into responders. Interestingly, co-delivery of K2-Fc slowed the IL-15/IL-15Rα-driven NK cell expansion down. We demonstrated earlier that IgG1 Fc-mediated effector functions suppress the expansion of NK and CD4^+^ T cells upon stimulation with recombinant IL-15^21^. We showed that K2-Fc reduced the amount of CD16 displaying NK cells. CD16 shedding occurs through ADAM17 upon NK cell activation and it has been shown recently, that activation of ADAM17 by IL-15 can limit human NK cell proliferation^41^. Our data suggest that ADCC might have a similar effect on IL-15-mediated NK cell expansion.

In contrast to most in vitro studies involving PBLs, we did not address the anti-tumor functions of NK and T cells individually but evaluated the interplay between CD4^+^ T cells, CD8^+^ T cells and NK cells on immune cell activation and tumor lysis upon delivery of IL-15/IL-15Rα and K2-Fc. We demonstrated that IL-15/IL-15Rα-mediated tumor killing follows distinct kinetics upon CD8^+^ T cell and NK cell depletion. While in presence of NK cells melanoma growth is immediately controlled, in their absence, CD8^+^ T cells allow tumor cell killing at a later time point. These findings are in concordance with the general understanding of how NK cells act as first-line defense and establish robust responses much quicker than CD8^+^ T cells^42^. Interestingly, the absence of CD8^+^ T cells led to a better tumor controle when no IL-15 was present. This could be explained by the nature of the assay where the tumor might become more accessible for NK cells when there is no steric hindrance through CD8^+^ T cells. However, in our experience information regarding in vitro kinetics of tumor cell killing is scarce. Although CD4^+^ T cells were dispensable for tumor lysis, we showed that CD4^+^ T cells influence NK cell activation and vice versa which may be important in vivo. Notaby, CD4^+^ T cell activation was considerably impaired in the absence of NK cells, while NK cells where only modestly impaired by CD4^+^ T cell depletion suggesting that CD4^+^ T cells might depend more on NK cells than the opposite is the case. CD4^+^ T cells and NK cells are major sources of respectively IL-2^43^ and IFN-γ^44^ and engage in a cross-talk that determines immune cell fate and orchestrates immune responses. However, interactions between CD4 and NK cells are poorly understood. It has been shown that CD134 expression on NK cells is transient and requires contact with T cells^45^. We confirm that, upon IL-15/IL-15Rα stimulation, CD134 expression on NK cells is transient and narrows down CD4^+^ T cells as T cell subset supporting CD134 induction on NK cells. Interestingly, K2-Fc-mediated upregulation of CD134 and CD137 on NK cells was strongly hampered in absence of CD4^+^ T cells, however, could enact in presence of IL-15/IL-15Rα and synergistically upregulate expression comparable with non-depleted or CD8^+^ T cell-depleted co-cultures. CD4^+^ T cells can act as helper and effector cells and have been investigated in the context of cytokine therapy^46^. We demonstrated that upon NK cell depletion, CD134 and CD137 expression on CD4^+^ T cells were strongly abrogated and IL-15/IL-15Rα could only marginally increase the expression, suggesting that NK cells are required for the activation of CD4^+^ T cells. Similarly, it has been shown that IFN-γ produced by NKT cells was essential for CD4^+^ T cell activation and antitumor function in a leukemia model^47^. Interestingly, neither CD4^+^ T cell nor NK cell depletion caused profound differences in CD8^+^ T cell activation, suggesting that allogeneic CD8^+^ T cell responses are independent on bystander cells. These data highlight the importance of studying the interactions between cells of innate and adaptive immunity when evaluating onco-immunological therapeutics.

Altogether, we demonstrated that employing a gene-based approach that could be used for intratumoral delivery of cytokine and immune checkpoint therapy is feasible and allows the induction and enhancement of anti-tumor immune responses. This is highly relevant given the increasing interest in combining IL-15 with anti-PD-L1 therapy and given the drawbacks of their systemic delivery as recombinant proteins (e.g., toxicity). Localized, intratumoral immunotherapy overcomes these issues. To date, intratumoral immunotherapy has been evaluated in accessible tumor types such as melanoma, priming the tumor for T cell-mediated rejection^48,49^. Notably, Intratumoral immunotherapy ensures high availability of the therapy agent at the local injection site, while restricting systemic exposure and off-target toxicity. Even though, the therapy agents’ availability is restricted, tumor responses in non-injected lesions have been observed in both preclinical and clinical studies following intratumoral immunotherapy^50,51^. Many therapy agents are under clinical investigation for intratumoral immunotherapy, including viral vectors such as oncolytic and non-oncolytic viruses, and nucleic acids such as mRNA^49,52^. However, more research is needed to identify the genetic vector that is best suited to translate the findings presented in this manuscript into a human setting. Given the significant interest in the development of novel delivery approaches for ICP drugs, it will undoubtedly be addressed in the near future which drug delivery systems could be utilized^53^.

## Conclusion

This study demonstrates that gene-based delivery of IL-15/IL-15Rα to tumor cells effectively mediates anti-tumor activity and sensitizes the tumor microenvironment for therapy with αPD-L1 therapeutics mainly by impacting NK cells. These findings warrant further investigation of gene-based IL-15 and K2-Fc delivery in vivo.

### Supplementary Information

Supplementary Information.

## Data Availability

The data in the current study are available from the corresponding authors upon reasonable request.

## Abbreviations

PD-1: Programmed cell death protein-1
PD-L1: Programmed death-ligand 1
PBL: Peripheral blood lymphocyte
IL: Interleukin
IFN: Interferon
Ig: Immunoglobulin
NK: Natural killer
TME: Tumor microenvironment
