# Supplementary material for: In vitro modelling of local gene therapy with IL-15/IL-15Rα and a PD-L1 antagonist in melanoma reveals an interplay between NK cells and CD4+ T cells
Source: Sci Rep. 2023 Nov 3;13:18995. doi: 10.1038/s41598-023-45948-w (PMC10624833; doi:10.1038/s41598-023-45948-w)
Supplement: Supplementary file 1 — Supplementary Information. [file 41598_2023_45948_MOESM1_ESM.docx]

**Supplementary Information**

Supplementary Figure 1:


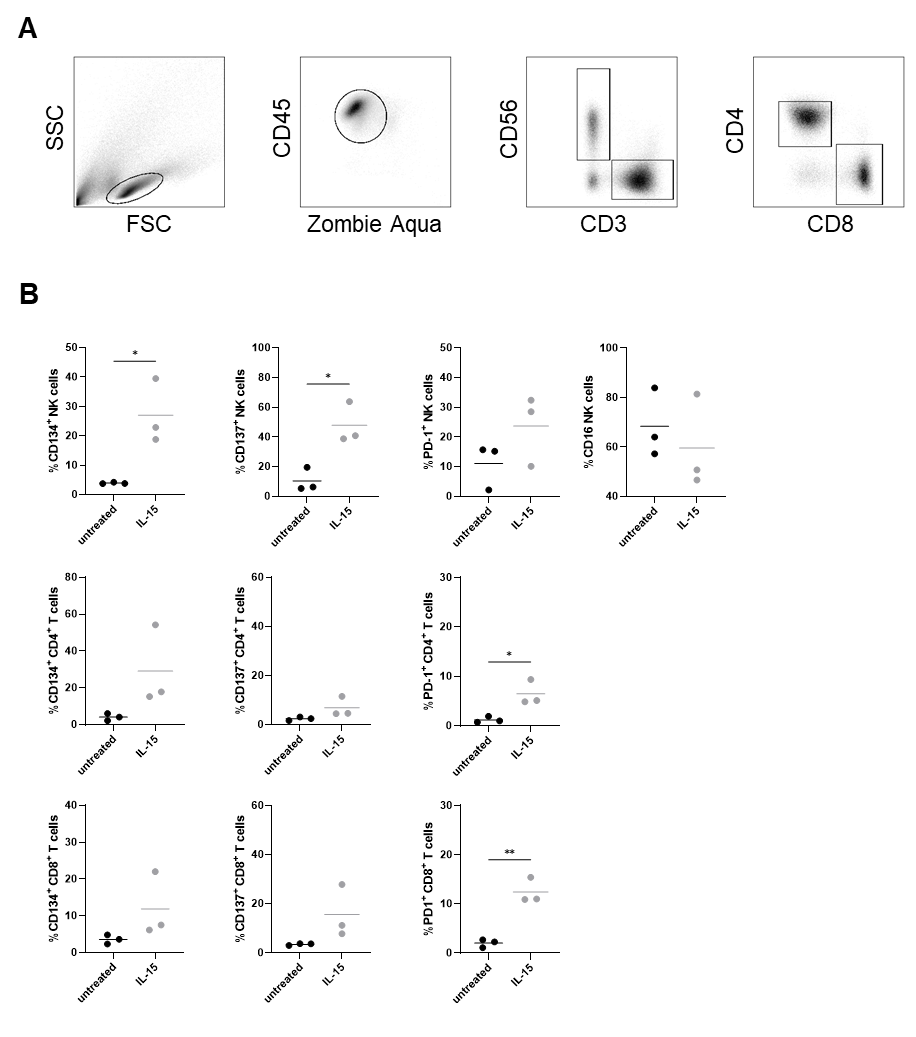


**Supplementary Figure 1: IL-15 modulates the immune cell activation.** (A) Gating strategy for discrimination of CD3^-^ CD56^+^ NK cells, CD3^+^ CD4^+^ T cells and CD3^+^ CD8^+^ T cells. (B) Expression of activation markers CD134 (left panel), CD137 (middle panel) and PD-1 (right panel) on CD3^-^ CD56^+^ NK cells (upper row), CD3^+^ CD4^+^ T cells (middle row) and CD3^+^ CD8^+^ T cells (lower row), measured in flow cytometry after 48 hours in co-culture.
